# APP‐derived peptides reflect neurodegeneration in frontotemporal dementia

**DOI:** 10.1002/acn3.50948

**Published:** 2019-12-02

**Authors:** Ignacio Illán‐Gala, Jordi Pegueroles, Victor Montal, Daniel Alcolea, Eduard Vilaplana, Alexandre Bejanin, Sergi Borrego‐Écija, Frederic Sampedro, Andrea Subirana, María‐Belén Sánchez‐Saudinós, Ricard Rojas‐García, Hugo Vanderstichele, Rafael Blesa, Jordi Clarimón, Anna Antonell, Albert Lladó, Raquel Sánchez‐Valle, Juan Fortea, Alberto Lleó

**Affiliations:** ^1^ Memory Unit Department of Neurology Hospital de la Santa Creu i Sant Pau Biomedical Research Institute Sant Pau Universitat Autònoma de Barcelona Barcelona Spain; ^2^ Centro de Investigación Biomédica en Red de Enfermedades Neurodegenerativas CIBERNED Madrid Spain; ^3^ Alzheimer’s Disease and Other Cognitive Disorders Neurology Department Hospital Clínic, Fundació Clínic per a la Recerca Biomèdica Institut d’Investigacions Biomèdiques August Pi I Sunyer University of Barcelona Barcelona Spain; ^4^ Movement Disorders Unit Neurology Department Hospital de la Santa Creu i Sant Pau Barcelona Spain; ^5^ Neuromuscular Diseases Unit Department of Neurology Hospital de la Santa Creu i Sant Pau Universitat Autònoma de Barcelona Barcelona Spain; ^6^ ADx NeuroSciences NV Gent Belgium; ^7^ Barcelona Down Medical Center Fundació Catalana de Síndrome de Down Barcelona Spain

## Abstract

**Objective:**

We aimed to investigate the relationship between cerebrospinal fluid levels (CSF) of amyloid precursor protein (APP)‐derived peptides related to the amyloidogenic pathway, cortical thickness, neuropsychological performance, and cortical gene expression profiles in frontotemporal lobar degeneration (FTLD)‐related syndromes, Alzheimer’s disease (AD), and healthy controls.

**Methods:**

We included 214 participants with CSF available recruited at two centers: 93 with FTLD‐related syndromes, 57 patients with AD, and 64 healthy controls. CSF levels of amyloid β (Aβ)1‐42, Aβ1‐40, Aβ1‐38, and soluble β fragment of APP (sAPPβ) were centrally analyzed. We compared CSF levels of APP‐derived peptides between groups and, we studied the correlation between CSF biomarkers, cortical thickness, and domain‐specific cognitive composites in each group. Then, we explored the relationship between cortical thickness, CSF levels of APP‐derived peptides, and regional gene expression profile using a brain‐wide regional gene expression data in combination with gene set enrichment analysis.

**Results:**

The CSF levels of Aβ1‐40, Aβ1‐38, and sAPPβ were lower in the FTLD‐related syndromes group than in the AD and healthy controls group. CSF levels of all APP‐derived peptides showed a positive correlation with cortical thickness and the executive cognitive composite in the FTLD‐related syndromes group but not in the healthy control or AD groups. In the cortical regions where we observed a significant association between cortical thickness and CSF levels of APP‐derived peptides, we found a reduced expression of genes related to synaptic function.

**Interpretation:**

APP‐derived peptides in CSF may reflect FTLD‐related neurodegeneration. This observation has important implications as Aβ1‐42 levels are considered an indirect biomarker of cerebral amyloidosis.

## Introduction

Frontotemporal lobar degeneration (FTLD) is a neuropathological umbrella term encompassing multiple proteinopathies with common patterns of neurodegeneration in frontotemporal regions.^1^ In contrast to Alzheimer’s disease (AD), there are no specific pathophysiological biomarkers for FTLD and current diagnostic criteria rely on the identification of particular syndromes and neurodegenerative patterns on neuroimaging.^1^ Cerebrospinal fluid (CSF) biomarkers have been studied in neurodegenerative disease as a way to track different pathophysiological processes in the central nervous system.^2^ Levels of core AD biomarkers, amyloid β1‐42 (Aβ1‐42), total Tau (t‐Tau), and phosphorylated Tau (p‐Tau), can be useful in FTLD‐related syndromes to exclude AD.^3^ Specifically, low Aβ1‐42 levels in CSF are considered a biomarker of cerebral amyloidosis in current research frameworks^4^ and therefore its presence in patients with FTLD‐related syndromes is usually interpreted as a sign of either atypical AD mimicking FTLD or comorbid AD pathology in FTLD cases.^5^

However, several studies in patients with FTLD have shown substantial disagreement between CSF levels of Aβ1‐42 and amyloid positron emission tomography.^6^, ^7^ In addition, previous studies have also reported lower CSF levels of Aβ1‐42 in pathology‐proven FTLD cases without comorbid AD,^3^ as well as in genetically confirmed FTLD,^8^ suggesting that in FTLD, Aβ1‐42 levels could be affected by mechanisms independent of AD pathology.^9^

Aβ peptides are generated by proteolytic cleavage from the amyloid precursor protein (APP) by sequential action of β‐ and γ‐secretases.^10^ In addition to Aβ1‐42, the proteolytic process of APP also generates other shorter Aβ species, such as Aβ1‐40 and Aβ1‐38, which can be used as markers of APP metabolism. Previous studies have reported that different Aβ peptides are reduced in FTLD‐related syndromes.^11^, ^12^, ^13^, ^14^ Another APP‐derived peptide generated from APP is the soluble β fragment of APP (sAPPβ).^10^, ^15^ sAPPβ is generated by BACE1, the main β‐secretase in the central nervous system, and levels are also reduced in CSF in FTLD‐related syndromes^11^, ^16^, ^17^, ^18^, ^19^, ^20^ and correlate with cortical thickness in frontotemporal regions.^18^, ^20^

However, it remains unknown whether Aβ1‐42 and other Aβ peptides generated in the amyloidogenic APP pathway are related to neurodegeneration in FTLD‐related syndromes or AD.^21^ We hypothesized that, similarly to what we have observed for sAPPβ, the decrease in the CSF levels of Aβ1‐42, Aβ1‐40, and Aβ1‐38 may reflect neurodegeneration in FTLD but not in AD, where amyloid‐related pathophysiological changes in APP processing may obscure this association.^22^ The identification of different patterns of APP‐derived peptides in CSF in FTLD and AD is important for the *in‐vivo* identification of FTLD and its differentiation from other non‐neurodegenerative diseases and could also provide information about central pathophysiological mechanisms in FTLD. In this multimodal biomarker study, we compare the CSF levels of Aβ1‐42, Aβ1‐40, Aβ1‐38, and sAPPβ in FTLD‐related syndromes, AD, and healthy controls, and we explore their relationship with cortical thickness and neuropsychological measures of cognitive impairment. To further explore the mechanisms underlying the relationship between APP‐derived peptides and neurodegeneration in FTLD, we investigated the transcriptional architecture of cerebral regions where cortical thickness correlated with APP‐derived peptides in the FTLD group.^23^

## Materials and Methods

### Study participants and classification

Patients with AD and FTLD‐related syndromes were diagnosed at each center by experts in behavioral neurology according to current diagnostic criteria.^24^, ^25^, ^26^, ^27^, ^28^ From 338 eligible participants with CSF available at both centers, we selected a subgroup of 219 participants (98 FTLD‐related syndromes, 57 AD, and 64 healthy controls) with a CSF sample stored in the same polypropylene tube. Moreover, to avoid the inclusion of FTLD‐related syndromes and healthy control participants with comorbid AD or atypical forms of AD that could alter the profile of APP‐derived peptides, we excluded five participants in the FTLD‐related syndromes group with a CSF biomarker profile suggestive of AD (as defined by low CSF levels of Aβ1‐42 levels and increased CSF levels of t‐Tau or p‐Tau) according to local validated thresholds at each center (916 and 500 pg/mL for Aβ1‐42; 456 and 450 pg/mL for t‐Tau and 63 and 75 pg/mL for p‐Tau, in Hospital de Sant Pau and HCP, respectively).^29^, ^30^, ^31^ In the AD group, we included only mild cognitive impairment and dementia of the Alzheimer’s type participants with low CSF levels of Aβ1‐42 levels and increased CSF levels of t‐Tau or p‐Tau using the same thresholds.^29^, ^30^, ^31^ Moreover, healthy controls included in this study did not have a CSF biomarker profile suggestive of preclinical AD using the same criteria and thresholds. For further details on sample composition, please refer to Supplementary material.

### Neuropsychological measures

Cognitive functioning was assessed using a previously described neuropsychological evaluation^32^ and Z‐scores were calculated for different cognitive domains as previously described (Supplementary material).^33^

### CSF sampling and analyses

All biomarkers were analyzed at the Sant Pau Memory Unit Laboratory with commercially available ELISA kits of Aβ1‐40, Aβ1‐38, and sAPPß (EUROIMMUN, Lübeck, Germany; IBL, Minneapolis, MN, respectively) following previously reported methods and manufacturer’s instructions.^30^, ^31^,^34^ To account for inter‐center variability in pre‐analytical protocols, we applied a previously validated harmonization method.^35^ For further details on CSF analyses, please refer to Supplementary material (Table [Link]).

### Image acquisition, processing, and analysis

A subset of 135 participants had 3T magnetic resonance imaging (MRI) available for quantitative neuroimaging analyses. Of these, 88 participants were scanned on a 3T Philips Achieva, 7 participants were scanned on a different 3T Philips Achieva, and 40 participants were scanned on 3T Siemens 3T TrioTim. Briefly, surface‐based cortical reconstruction was performed using FreeSurfer v5.1 software package (http://surfer.nmr.mgh.harvard.edu) to compute individual cortical thickness maps, as previously reported.^36^ Each individual cortical thickness map was morphed to Freesurfer’s standard surface space and smoothed applying a Gaussian kernel of 15 mm full width at half maximum as customary in surface‐based analyses. Regarding neuroimaging analyses, a vertex‐wise general linear model (as implemented in FreeSurfer v5.1; Athinoula A. Martinos Center for Biomedical Imaging, Charlestown, MA, USA) was used to assess the correlation between CSF biomarkers and cortical thickness for each group independently. Specifically, for each surface vertex, a general linear model was computed using cortical thickness as dependent variable and CSF values as independent variable. All these analyses were adjusted by age, sex, and magnetic resonance equipment. To control for false positives, a Monte‐Carlo simulation with 10000 repeats as implemented in FreeSurfer (FWE <0.05) was tested.

### Genetic studies

*APOE* was genotyped in all participants according to previously described methods.^37^ Participants in the FTLD‐related syndromes group were screened for most common FTLD‐related mutations as previously described.^20^

### Statistical methods

We assessed normality of the variables by means of the Kolmogorov–Smirnov test. Between‐group differences in baseline characteristics were assessed using t‐test, ANOVA, or Kruskal–Wallis test for continuous variables, and χ^2^ for categorical data. Between‐group differences in CSF biomarkers were assessed with robust ANOVA (F welch) and analysis of covariance (ANCOVA) analyses with age and *APOE*ɛ4 status as covariates. For ANOVA and ANCOVA analyses, variables not following a normal distribution (Aβ1‐42, Aβ1‐38, and sAPPβ) were log‐transformed to achieve normality. For the study of the correlations within CSF biomarkers and between CSF biomarkers and cognitive measures, we applied simple correlations (Pearson’s coefficient) and partial correlations (including age, sex, and education as covariates for cognitive measures) with bootstrapping‐based 95% confidence intervals (bias corrected and accelerated for 1000 samples). To explore the significant differences between the magnitude of the correlations of CSF biomarkers between groups, we applied a R‐to‐R transform. All statistical tests were two‐sided and statistical significance was set at 5% (α = 0.05). Statistical analyses were performed with the IBM SPSS Statistics 25 (IBM corp.) software. For ANOVA and ANCOVA analyses, all *P* values were corrected for multiple comparisons (Bonferroni).

### Cortical gene expression

Microarray data from a total of 3702 regional tissue sample from post‐mortem brain tissue of six individuals from the Allen Institute Human Brain Atlas were used to analyze cortical gene expression (http://human.brain-map.org/). Data were processed according to previously published methods (for more details, see Supplementary material).^38^

### Gene set enrichment analysis

After the identification of cortical regions where cortical thickness showed a significant correlation with the CSF levels of APP‐derived peptides, we quantified the differences in gene expression between these areas and the background expression levels in the rest of the cortex, calculating the Cohen’s delta for each gene. The resulting Cohen’s deltas were first normalized using Z‐score and then used to build a histogram distribution of the differences between ROI and background expression. Then, a threshold of 2.58 × SD (99% confidence interval) was set to obtain the list of genes showing a differential expression. Then, we introduced these genes in the GO Enrichment Analysis^39^, ^40^ with Fisher’s exact test and false discovery rate correction to perform the enrichment analysis. To test whether genes interacting with APP were overexpressed in the cortical regions, we evaluated the genes encoding for proteins with the highest confidence score of interaction from STRING (https://string-db.org/).

### Standard protocol approvals, registrations, and patient consent

The study was approved by the local ethics committee of each participant center and was conducted in accordance with the Declaration of Helsinki. All participants gave their written informed consent to participate in the study.

### Data availability

The datasets analyzed during the current study are available from the corresponding author on reasonable request.

## Results

### Demographics and clinical characteristics of participants

The main clinical and demographic characteristics of the study are shown in Table 1. There were no differences in age at CSF sampling and MMSE score between the FTLD‐related syndromes and AD groups. However, the FTLD‐related syndromes group was more functionally impaired and showed a higher frequency of men than the AD group. The healthy control group was younger at CSF sampling than the FTLD‐related syndromes and AD groups. As expected, *APOE*ɛ4 carriers were overrepresented in the AD group compared to FTLD‐related syndromes and controls. In the FTLD‐related syndromes group, there were 11 cases with known pathogenic mutations in FTLD‐related genes (6 *C9orf72*, 4 *GRN*, and 1 *VCP*).

**Table 1:** Demographic, clinical, and neuropsychological data.

Demographic, clinical and genetic characteristics	FTLD‐related syndromes	Alzheimer’s disease	Healthy controls	Statistics and *P* values
*n* participants (HSP/HCB)	93 (76/17)^ns^	57 (45/12)^ns^	64 (53/11)^ns^	χ^2^(2) = 0.3 *P* = 0.854
Age at CSF, years, median (Q1, Q3)	70.7 (64.7, 77.4)^1^	72.3 (69.3, 76.5)^1^	64.3 (61, 69.6)^2^, ^3^	*F*(2, 214) = 14.2 ***P* < 0.001**
Age at clinical onset, years, median (Q1, Q3)	66 (60, 72)^3^	69.5 (65, 73)^2^	–	*t*(144) = 39.8 ***P* = 0.001**
Male, *n* (%)	54 (58)^3^	21 (37)^2^	31 (48)	χ^2^(2) = 6.4 ***P* = 0.041**
*APOE*ɛ4+, *n* (%)	18 (19)^3^	29 (51)^1^, ^2^	12 (19)^3^	χ^2^(2) = 21.1 ***P* < 0.001**
Education, years, median (Q1, Q3)	11 (8, 15)^ns^	9 (8, 13)^1^	15 (9, 18)^3^	*H*(2) = 12.9 ***P* = 0.003**
MMSE score, median (Q1, Q3)	26 (21.5, 28)^1^	25 (19.5, 27)^1^	29 (28, 30)^2^, ^3^	*H*(2) = 62.2 ***P* < 0.001**
IDDD, total score, median (Q1, Q3)	44 (39, 57)^1^, ^3^	38 (33, 47)^1^, ^2^	33 (33, 33)^2^, ^3^	*H*(2) = 72.9 ***P* < 0.001**
*n* 3T MRI available (HSP/HCB)	54 (37/17)^ns^	38 (26/12)^ns^	43 (32/11)^ns^	χ^2^(2) = 0.5 *P* = 0.780
Neuropsychological evaluation available, *n* (%)	63 (68)^ns^	42 (74)^ns^	53 (83)^ns^	χ^2^(2) = 4.5 *P* = 0.108
Memory z‐score, median (Q1, Q3)	−3.6 (−6.4, −1.7)^1^, ^3^	−5.2 (−7.0, −3.6)^1^, ^2^	0 (−0.4, 0.5)^2^, ^3^	*H*(2) = 99.3 ***P < 0.001***
Executive z‐score, median (Q1, Q3)	−1.7 (−2.7, −0.9)^1^	−1.5 (−2.3, −0.7)^1^	0 (−0.4, 0.4)^2^, ^3^	*H*(2) = 62.7 ***P < 0.001***
Language z‐score, median (Q1, Q3)	−2.1 (−2.9, −1.2)^1^	−2.2 (−3.1, −1.3)^1^	0 (−0.4, 0.4)^2^, ^3^	*H*(2) = 79.9 ***P < 0.001***
Visuospatial z‐score, median (Q1, Q3)	−1.7 (−3.5, −0.4)^1^	−1.3 (−2.5, −0.1)^1^	0 (−0.1, 0.5)^2^, ^3^	*H*(2) = 54.9 ***P < 0.001***

Statistically significant results are bold. CSF, cerebrospinal fluid; FTLD, frontotemporal lobar degeneration; HSP, Hospital de Sant Pau; HCB, Hospital Clínic de Barcelona; IDDD, interview for deterioration in daily living activities in dementia; MMSE, mini‐mental state examination; MRI, magnetic resonance imaging; *n*, number; Q1, first quartile; Q3, third quartile; 3T, 3 tesla.

^1^ Different than the healthy control group (*P* < 0.05).

^2^ Different than the FTLD‐related syndromes group (*P* < 0.05).

^3^ Different than the Alzheimer’s disease group (*P* < 0.05).

### CSF levels of APP‐derived peptides are reduced in FTLD‐related syndromes

**Figure 1:**
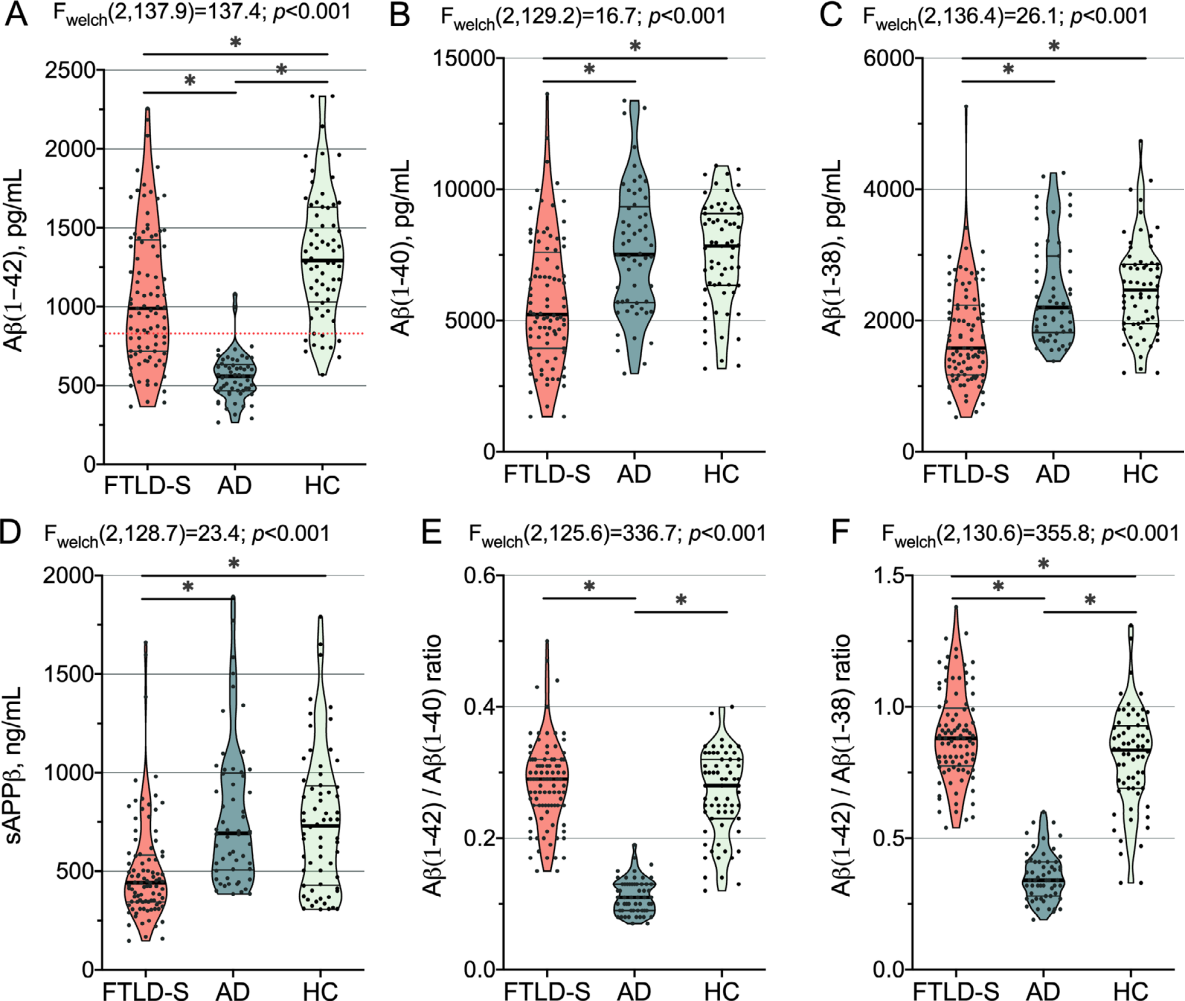
Group comparison of CSF levels of APP‐derived peptides. CSF levels of (A) Aβ1‐42, (B) Aβ1‐40, (C) Aβ1‐38, (D) sAPPβ, (E) Aβ1‐42/Aβ1‐40 ratio, and (F) Aβ1‐42/Aβ1‐38 ratio across groups. Only statistically significant differences are displayed. We applied correction for multiple comparisons (Bonferroni’s post‐hoc test; *P < 0.001). The red‐dotted line represents our previously validated CSF cut‐point for Aβ1‐42. AD, Alzheimer’s disease; Aβ, amyloid β; FTLD‐S, frontotemporal lobar degeneration‐related syndromes; HC, healthy controls; sAPPβ, soluble β fragment of amyloid precursor protein; CSF, cerebrospinal fluid; APP, amyloid precursor protein.

CSF levels of Aβ1‐42, Aβ1‐40, Aβ1‐38, and sAPPβ were reduced in the FTLD‐related syndromes group when compared to the healthy control group (Fig. 1A–D). As shown in Table 2, the CSF levels of Aβ1‐42 were significantly different between groups even after accounting for age at CSF sampling and the presence of *APOE*ɛ4. As expected, the lowest levels of Aβ1‐42 were observed in the AD group. However, Aβ1‐42 levels were also decreased in FTLD‐related syndromes when compared to healthy controls.

**Table 2:** Cerebrospinal fluid biomarker data.

CSF biomarkers	FTLD‐related syndromes	Alzheimer’s disease	Healthy controls	ANCOVA^1^
Aβ1‐42, pg/mL^2^, median (Q1, Q3)	989 (718, 1423)^3^, ^4^	560 (467, 634)^4^, ^5^	1294 (1030, 1631)^3^, ^5^	*F*(2, 209) = 72.4 ***P* < 0.001** partial η^2^ = 0.409
Aβ1‐40, pg/mL, median (Q1, Q3)	5224 (3937, 7593)^3^, ^4^	7511 (5686, 9339)^5^	7842 (6341, 9075)^5^	*F*(2, 208) = 17.5 ***P* < 0.001** partial η^2^ = 0.144
Aβ1‐38, pg/mL^2^, median (Q1, Q3)	1582 (1172, 2235)^3^, ^4^	2199 (1817, 2983)^5^	2464 (1953, 2856)^5^	*F*(2, 209) = 29.6 ***P* < 0.001** partial η^2^ = 0.221
sAPPβ, ng/mL^2^, median (Q1, Q3)	442 (345, 582)^3^, ^4^	693 (507, 998)^5^	731 (429, 934)^5^	*F*(2, 209) = 24.7 ***P* < 0.001** partial η^2^ = 0.191
Aβ1‐42/Aβ1‐40 ratio median (Q1, Q3)	0.29 (0.25, 0.32)^3^	0.11 (0.09, 0.13)^4^, ^5^	0.28 (0.23, 0.32)^3^	*F*(2, 208) = 133.1 ***P* < 0.001** partial η^2^ = 0.561
Aβ1‐42/Aβ1‐38 ratio, median (Q1, Q3)	0.88 (0.79, 1.00)^3^, ^4^	0.34 (0.28, 0.41)^4^, ^5^	0.83 (0.69, 0.93)^3^, ^5^	*F*(2, 207) = 149.7 ***P* < 0.001** partial η^2^ = 0.591

Statistically significant results are bold. ANCOVA, analysis of covariance; Aβ, amyloid β; CSF, cerebrospinal fluid; FTLD, frontotemporal lobar degeneration; sAPPβ, soluble β fragment of amyloid precursor protein.

^1^ ANCOVA adjusted for age at CSF sampling and *APOEɛ4* status.

^2^ These variables were not normally distributed across groups and were log‐transformed to achieve normality before the statistical analyses.

^3^ Different than the Alzheimer’s disease group (*P* < 0.001; Bonferroni pairwise comparisons with bias‐corrected accelerated bootstrapping for 1000 samples).

^4^ Different than the healthy control group (*P* < 0.001; Bonferroni pairwise comparisons with bias‐corrected accelerated bootstrapping for 1000 samples).

^5^ Different than the FTLD‐related syndromes group (*P* < 0.001; Bonferroni pairwise comparisons with bias‐corrected accelerated bootstrapping for 1000 samples).

As reported in previous studies,^6^, ^7^ in a substantial proportion of FTLD‐related syndromes, participants (*n* = 40; 43% of the FTLD‐related syndromes group) had low CSF levels of Aβ1‐42, below our validated threshold for cerebral amyloidosis.^31^ However, only 13 participants in the frontotemporal lobar degeneration‐related syndromes (FTLD‐S) group (17%) had higher CSF levels of either t‐Tau or p‐Tau and normal levels of Aβ1‐42. Importantly, we observed the same results when we excluded these cases from the FTLD‐S group.

The CSF levels of Aβ1‐40, Aβ1‐38, and sAPPβ also differed between groups after accounting for age at CSF sampling and the presence of *APOE*ɛ4. The CSF levels of Aβ1‐40, Aβ1‐38, and sAPPβ were lower in the FTLD‐related syndromes group than in the AD and healthy control groups (Fig. 1B–D). As expected, the Aβ1‐42/Aβ1‐40 and Aβ1‐42/Aβ1‐38 ratios differed between groups (Fig. 1E–F). The Aβ1‐42/Aβ1‐40 and the Aβ1‐42/Aβ1‐38 ratios were lower in the AD group than in the FTLD‐related syndromes and healthy control groups. The Aβ1‐42/Aβ1‐38 ratio was higher in the FTLD‐related syndromes group than in the healthy control group. We did not find significant differences in the CSF levels of APP‐derived peptides between FTLD‐related syndromes clinical subgroups.

We did not find any significant differences in the CSF levels of APP‐derived peptides between participants with increased certainty of underlying FTLD‐Tau (*n* = 30, progressive supranuclear palsy‐corticobasal degeneration spectrum [PSP‐CBD], and non‐fluent variant of primary progressive aphasia [nfaPPA] participants) and FTLD‐TDP (*n* = 21, including patients with semantic variant PPA, patients with motor neuron disease and carriers of *C9orf72*, *GRN*, and *VCP* mutations). However, patients with *C9orf72*, *GRN*, and *VCP* mutations (*n* = 11) had lower levels of Aβ1‐42 than patients in the sporadic FTLD‐S group (*n* = 82; t(18.2) = 2.158; *P* = 0.045).

Of note, we did not observe statistically significant differences in the CSF levels of APP‐derived peptides between *APOE*ɛ4 positive (*n* = 18) and *APOE*ɛ4 negative (*n* = 75) FTLD‐S cases. This finding together with the similar Aβ1‐42/Aβ1‐40 ratio in FTLD‐S cases and controls suggests that comorbid AD may not be the cause of the observed decrease in APP‐derived peptides in the FTLD‐S group.

### APP‐derived peptides correlate with cortical thickness in FTLD‐related syndromes

**Figure 2:**
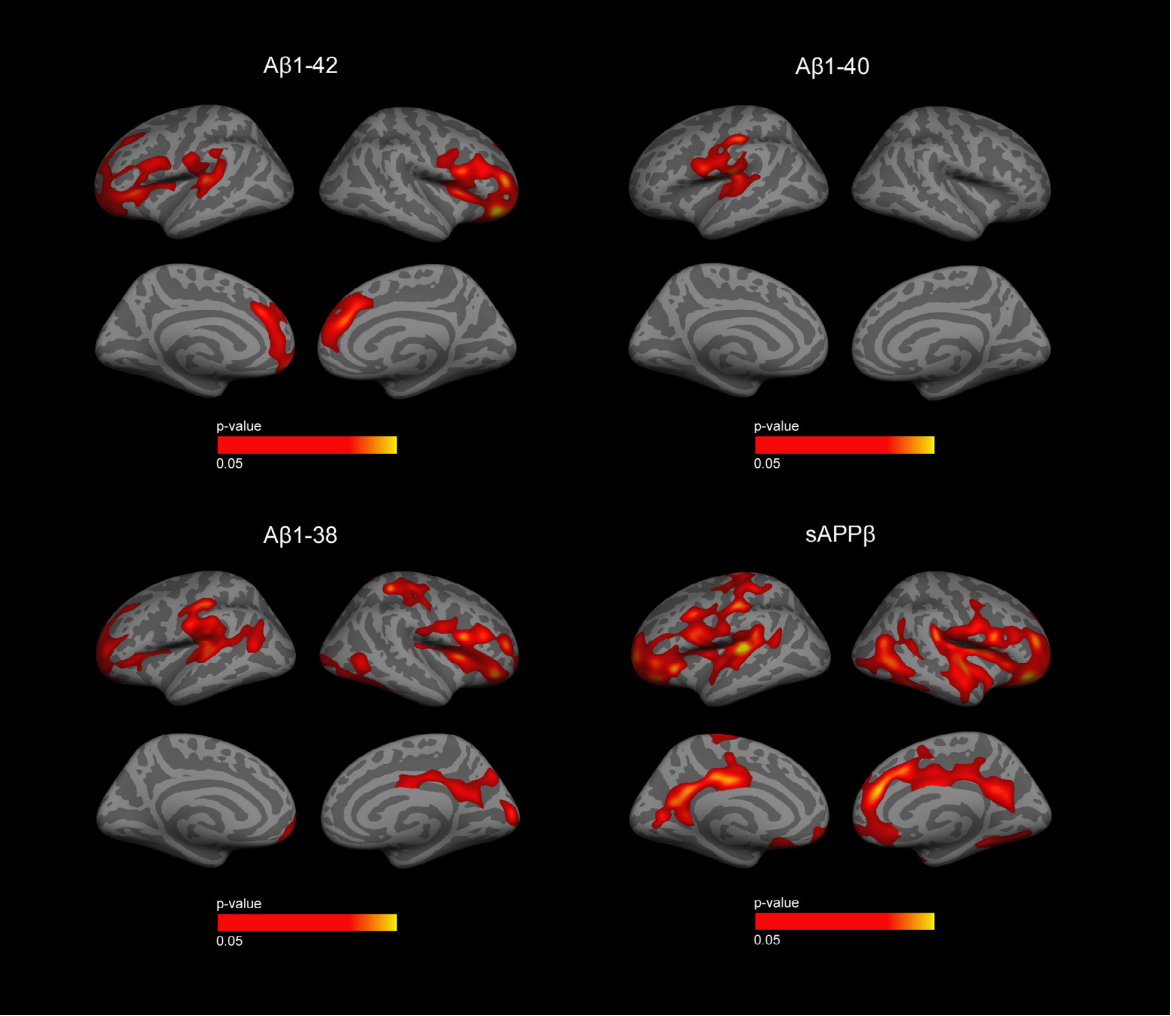
Correlation of APP‐derived peptides with cortical thickness in the FTLD‐related syndromes group. Correlation of APP‐derived peptides with cortical thickness in the FTLD‐related syndromes group. Only clusters that survived family‐wise error correction *P* < 0.05 are shown. Only positive correlations were found (displayed in orange and red). Cortical thickness analyses were adjusted for age, sex, and MRI equipment. Aβ, Amyloid β; sAPPβ, soluble β fragment of amyloid precursor protein; APP, amyloid precursor protein; FTLD‐S, frontotemporal lobar degeneration‐related syndromes; MRI, magnetic resonance imaging.

As shown in Figure 2, we found a positive correlation between APP‐derived peptides and cortical thickness in the FTLD‐related syndromes group. Particularly, the CSF levels of Aβ1‐42 showed a positive correlation with cortical thickness (namely, lower CSF values of Aβ1‐42 reflected cortical thinning) in the prefrontal cortex, insula, anterior and dorsal cingulate, and superior frontal cortex in both hemispheres. The CSF levels of Aβ1‐40 showed a positive correlation with cortical thickness in the inferior perirolandic cortex, superior temporal and insula, but this correlation was restricted to the left hemisphere. The CSF levels of Aβ1‐38 showed a positive correlation with cortical thickness in the prefrontal cortex, insula, and perirolandic cortex of both hemispheres (Fig. 2). Finally, the CSF levels of sAPPβ also showed widespread positive correlation with cortical thickness along the prefrontal cortex, insula, anterior and dorsal cingulate, perirolandic cortex, but also posterior cortical regions such as the precuneus (Fig. 2). We also explored the correlation of APP‐derived peptides in CSF in the different clinical subgroups of FTLD‐related syndromes. We obtained similar results for the behavioral variant of frontotemporal dementia subgroup with MRI available (*n* = 28). We also found significant correlations in the semantic variant of primary progressive aphasia group despite the small sample size (*n* = 5). However, we were unable to obtain significant correlations in the nfaPPA (*n* = 14) and PSP‐CBS (*n* = 5) subgroups. Of note, these two subgroups were the ones with the lowest neurodegenerative burden. Importantly, we did not observe any correlation between APP‐derived peptides and cortical thickness in the AD and healthy control groups despite the existence of extensive areas of cortical thinning in regions related with neurodegeneration in both groups (data not shown). Of note, neither Aβ1‐42/Aβ1‐40 nor Aβ1‐42/Aβ1‐38 ratios correlated with cortical thickness in the FTLD‐related syndromes or the AD groups.

### APP‐derived peptides relate to measures of cognitive function in FTLD‐related syndromes

We next investigated the correlation between APP‐derived peptides and domain‐specific cognitive scores in each clinical group. Importantly, we only found correlations in the FTLD‐related syndromes group, and these correlations remained significant after accounting for age, sex, and education. As shown in Table 3, the executive function composite showed a significant correlation with Aβ1‐42, Aβ1‐40, Aβ1‐38, and sAPPβ and the visuospatial function cognitive composite correlated with Aβ1‐42 and Aβ1‐38.

**Table 3:** Partial correlations of cognitive composites with APP‐derived CSF biomarkers after adjusting by age, sex, and education in the FTLD‐related syndromes group.

	Aβ1‐42	Aβ1‐40	Aβ1‐38	sAPPβ
Memory	n.s	n.s	n.s	n.s
Executive function	**0.41 (0.19–0.61)** ***P* = 0.001** *	**0.31 (0.05–0.56) *P* = 0.017**	**0.35 (0.12–0.57) *P* = 0.006**	**0.34 (0.13–0.53) *P* = 0.007**
Language	n.s	n.s	n.s	n.s
Visuospatial	**0.34 (0.09–0.56)** ***P* = 0.009**	n.s	**0.37 (0.13–0.58) *P* = 0.004**	n.s

Correlation between Aβ(1‐42), Aβ(1‐40), and Aβ(1‐38) and sAPPβ in (A) AD group, (B) FTLD‐related syndromes group, and (C) healthy control group. Results are shown as Pearson correlation coefficient (95% confidence interval). 95% confidence intervals were calculated by means of bias‐corrected accelerated bootstrapping (1000 samples). Significant correlations (*P* < 0.05) are marked in bold. APP, amyloid precursor protein; Aβ, amyloid β; CSF, cerebrospinal fluid; FTLD, frontotemporal lobar degeneration; n.s, non‐significant correlation.

* Remained significant after adjusting for multiple comparisons (Bonferroni).

### The correlation between different APP‐derived peptides varied across groups

We next explored whether the correlation between different APP‐derived peptides differed between groups. As shown in Table 4, correlations between peptides were in general stronger in the FTLD group compared to the other two groups. The CSF levels of sAPPβ showed moderate correlations with Aβ1‐42, Aβ1‐40, and Aβ1‐38 in FTLD‐related syndromes and control groups but not in the AD group, where only mild correlations were observed. sAPPβ levels did not correlate with Aβ1‐38 levels in the AD group. Comparison of Pearson coefficient values showed that the correlation of CSF levels of Aβ1‐42 with Aβ1‐38 and sAPPβ was higher in FTLD‐related syndromes group than in the AD group. The correlation of the CSF levels of Aβ1‐42 with Aβ1‐40 and Aβ1‐38 was higher in the FTLD‐related syndromes group than in the healthy control group.

**Table 4:** Correlations between different APP‐derived peptides across clinical groups.

	Aβ1‐42	Aβ1‐40	Aβ1‐38
Alzheimer’s disease			
sAPPβ	0.29 (0.04–0.52)^b^	0.39 (0.14–0.59)	0.37 (0.14–0.58)
Aβ1‐42	–	**0.70 (0.56–0.81)**	**0.59 (0.40–0.73)** ^b^
Aβ1‐40	–	–	**0.82 (0.73–0.89)**
FTLD‐related syndromes			
sAPPβ	**0.60 (0.38–0.77)** ^a^	**0.58 (0.35–0.73)**	**0.60 (0.42–0.64)**
Aβ1‐42	–	**0.84 (0.77–0.90)** ^c^	**0.89 (0.84–0.93)** ^ac^
Aβ1‐40	–	–	**0.88 (0.82–0.92)**
Healthy control			
sAPPβ	0.42 (0.18**–**0.63)	0.47 (0.25**–**0.65)	**0.57 (0.37–0.72)**
Aβ1‐42	–	**0.60 (0.40–0.76)** ^b^	**0.62 (0.40–0.80)** ^b^
Aβ1‐40	–	**–**	**0.89 (0.82–0.94)**

Correlation between Aβ1‐42, Aβ1‐40, and Aβ1‐38 and sAPPβ in (A) AD group, (B) FTLD‐related syndromes group, and (C) healthy control group. Results are shown as Pearson’s correlation coefficient (95% confidence interval). 95% confidence intervals were calculated by means of bias‐corrected accelerated bootstrapping (1000 samples). Moderate‐to‐high correlations (*r* > 0.5) are in bold. The correlations that are significantly different between groups are underlined; a: different from the AD group; b: different from the FTLD‐related syndromes group; c: different from the healthy control group. APP, amyloid precursor protein; Aβ, amyloid β; FTLD, frontotemporal lobar degeneration; sAPPβ, soluble β fragment of amyloid precursor protein.

### Transcriptional and gene set enrichment analysis

**Figure 3:**
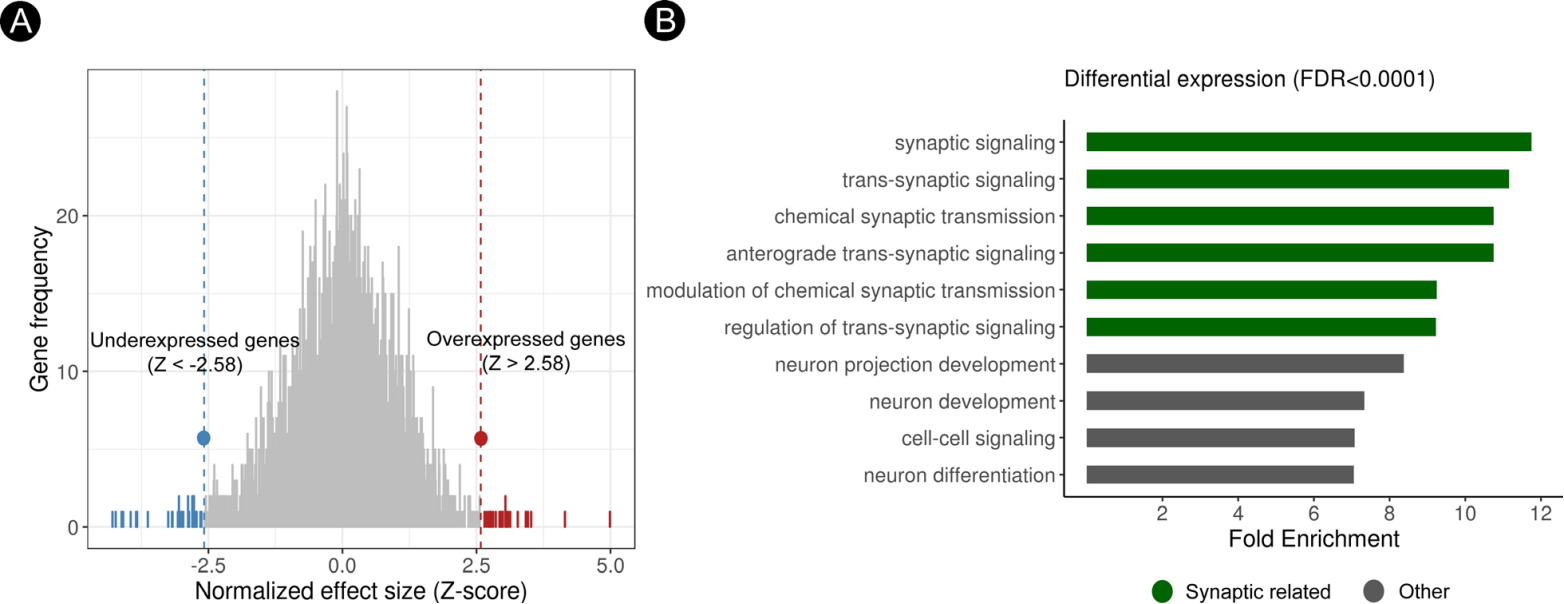
Transcriptional architecture and gene set enrichment analysis. (A) Histogram representing the distribution of the effect size scores of the neuro‐related gene expressions between regions with and without a significant relationship with APP‐derived peptides in FTLD. The full list of genes with differential expression can be found in Table S2. (B) Bar graphs show the top FDR‐corrected (*P* < 0.0001) GO enrichment analyses of genes differential expression in the brain regions where correlations were observed. APP, amyloid precursor protein; FTLD‐S, frontotemporal lobar degeneration‐related syndromes; FDR, false discovery rate.

To further investigate the potential mechanisms underlying the reduction of APP‐derived peptides in FTLD, we explored whether *APP* and other genes related to APP processing were overexpressed in the cortical regions with significant associations. We found that neither *APP* nor other *APP*‐related genes (for further details see Table S2) were differentially expressed in the cortical regions with positive correlations in the FTLD‐related syndromes group (Fig. 3A and Table S3). Finally, we investigated the transcriptional architecture of cerebral regions where cortical thickness showed a significant correlation with APP‐derived peptides. As shown in Figure 3B, in these regions, we observed a differential expression of genes related to synaptic processes, neuronal differentiation, and neurogenesis (Table S4). Of note, among genes with a differential expression, we found some genes codifying for synaptic proteins such as *NPTX1* and *CPLX2* (Table S2).

## Discussion

In this study, we describe for the first time a direct correlation between CSF levels of Aβ1‐42, Aβ1‐40, Aβ1‐38, and sAPPβ peptides with cortical thickness and neuropsychological measures in FTLD‐related syndromes. Our findings extend data from previous studies that had related APP processing and FTLD^17^, ^41^, ^42^, ^43^ and suggest that APP‐derived peptides are involved in FTLD through amyloid‐independent mechanisms.^10^ In addition, our transcriptional analyses suggested that biological processes related to synaptic function could link neurodegeneration and APP processing in FTLD.

Our findings that Aβ1‐42 levels are reduced in FTLD have important implications for the field of CSF biomarkers as Aβ1‐42 levels are considered an indirect biomarker of cerebral amyloidosis in current AD research frameworks.^4^, ^44^ Our results suggest that CSF levels of Aβ1‐42 could be reflecting two distinct but related phenomena: on the one hand, Aβ1‐42 levels are known to be associated with amyloid cerebral deposition, but on the other hand, Aβ1‐42 could be also related to synaptic loss in FTLD and possibly other non‐AD neurodegenerative diseases. Overall, the notion that CSF levels of APP‐derived peptides (including Aβ1‐42) may be also reduced in diseases other than AD argues against the role of Aβ1‐42 levels in CSF as a measure of cerebral amyloidosis only and suggests that it may also reflect other pathophysiological processes (i.e., synaptic loss or neuroinflammation). This is supported by previous reports in several neurodegenerative and neuroinflammatory conditions. For example, low CSF Aβ1‐42 levels have been related to disease severity and clinical progression in multiple sclerosis^45^ and Lewy body disease (Parkinson’s disease and Lewy body dementia).^46^ In addition, the CSF levels of Aβ1‐42 have been found decreased in neuropathologically confirmed cases of Creutzfeldt–Jakob disease independently of cerebral amyloid deposition^47^ and in neuroinfectious diseases.^48^ This evidence supports the view that the CSF levels of APP‐derived peptides could reflect pathophysiological mechanisms other than cerebral amyloid deposition.^10^

This study also helps to explain the improved agreement between amyloid PET and CSF measures observed with the Aβ1‐42/Aβ1‐40 ratio in non‐AD dementias.^6^, ^7^ Because both Aβ1‐40 and Aβ1‐38 are reduced in FTLD but not in AD, the Aβ1‐42/Aβ1‐40 and Aβ1‐42/Aβ1‐38 ratios can correct for the effect of FTLD‐related neurodegeneration on the CSF levels of APP‐derived peptides yielding a measure that is more specifically linked to cerebral amyloid deposition than levels of Aβ1‐42 alone.

We found a direct correlation between the CSF levels of Aβ peptides and cortical thickness in FTLD‐related syndromes but not in AD. Our results expand previous findings that sAPPβ correlate with cortical thickness in FTLD,^18^, ^20^, ^49^ by showing correlation also with Aβ1‐42, Aβ1‐40, and Aβ1‐38. In line with this result, we also found that CSF levels of APP‐derived peptides correlated with neuropsychological measures in the FTLD‐related syndromes group. The association between APP‐derived peptides and cortical thickness suggests that these peptides act as neurodegeneration biomarkers in FTLD. This hypothesis is consistent with a recent study showing that CSF levels of Aβ1‐42 correlated with gray matter density and were comparable to neurofilament light as a predictor of longitudinal MRI changes in FTLD‐related syndromes.^43^ Further studies are needed to determine the role of APP‐derived peptides as diagnostic and prognostic biomarkers in FTLD as well as their added value in comparison to other biomarkers such as neurofilament light or the p‐Tau/t‐Tau ratio.^50^

The lack of correlation between APP‐derived peptides and cortical thickness in the AD group deserves further discussion. If CSF levels of APP‐derived peptides reflect also neurodegeneration, then it would be expected to find a relationship between CSF levels, cortical thickness, and cognitive measures in the AD group. Several factors could explain why we failed to find these correlations in our study. First, the effect of cerebral amyloid plaque deposition on the dynamics of production and clearance of Aβ to the CSF may obscure a correlation with cortical thickness in the AD group. Additionally, we and others have shown that β‐site amyloid precursor protein‐cleaving enzyme (BACE) activity is increased in sporadic AD,^22^, ^51^ which may lead to a compensatory increase in APP‐derived peptides. Finally, it is possible that our relatively small AD group do not accurately reflect the heterogeneity of AD neurodegeneration and that only a subset of cases show lower levels of APP‐derived peptides in CSF.^52^ Indeed, one large multicenter study reported that the data‐driven subgroup of AD characterized by a diffuse pattern of atrophy had the lowest levels of Aβ1‐42 in CSF.^52^ This effect could mask a neurodegeneration‐mediated reduction of APP‐derived peptides with the exception of Aβ1‐42 which would be reduced due to sequestration in amyloid plaques.^2^ Taken together, as suggested in recent studies,^21^, ^52^ it remains to be elucidated whether CSF levels of Aβ1‐42 may also reflect neurodegeneration in AD in addition to cerebral amyloid deposition.

The transcriptional analyses performed in this study suggest that biological processes related to synaptic function could link neurodegeneration and APP processing in FTLD. Interestingly, there is strong evidence that APP is expressed at the synapse and that APP‐derived peptides are involved in multiple synaptic functions.^10^ Further links between neuronal activity and synaptic function of APP‐derived peptides come from studies showing that Aβ levels in CSF fluctuate over time in the same individuals under physiological conditions, and a reduction of Aβ production during sleep has been related to decreased cerebral activity.^53^ Taken together, previous evidence combined with our data support the idea that the reduction of APP‐derived peptides in CSF reflects synaptic dysfunction in FTLD and more studies are warranted to determine the underlying mechanisms of our findings.

The main strengths of this study are the inclusion of participants with a high probability of underlying FTLD and AD and the multimodal biomarker approach that allowed us to explore the structural correlates of APP‐derived peptides and their transcriptional architecture. However, this study has also some limitations. First, this study lacks neuropathological confirmation and misdiagnosis could have occurred. However, in all participants, the diagnosis of underlying FTLD and AD was supported by CSF cutoffs and longitudinal follow‐up. Although we did not find differences in APP‐derived peptides between specific clinical syndromes related to FTLD or participants with presumed Tau and TDP‐43 pathology in our study, further path‐confirmed studies should compare the CSF profile of APP‐derived peptides between the different neuropathological subtypes of FTLD. Moreover, additional studies should compare genetic and sporadic cases since results from this and other studies suggest the existence of different CSF biomarker profiles in genetic cases.^5^, ^54^, ^55^ Second, we relied on cross‐sectional data and thus, we were not able to assess the impact of APP‐related peptides in longitudinal clinical trajectories. Thus, more studies are needed to evaluate whether APP‐derived peptides can predict changes in biomarker trajectories and clinical outcomes of FTLD‐related neurodegeneration. Finally, although we used CSF criteria to exclude patients with FTLD‐related syndromes that had a CSF biomarker profile of AD, we cannot exclude entirely that some of the FTLD‐related syndromes may have some concurrent AD pathology and therefore this study could not account for comorbid AD pathology. Finally, the exploratory transcriptional analyses were performed in subjects without neurodegeneration and further studies in brain samples from subjects with FTLD are needed to confirm the underlying biological processes of this association.

In summary, we report that patients with FTLD show a global reduction of APP‐derived peptides in CSF and that this reduction correlated with FTLD‐related neurodegeneration and cognitive impairment. This pattern of APP‐derived peptides should be considered when evaluating the presence of AD pathophysiology in FTLD‐related syndromes. More studies are needed to advance our mechanistic understanding of the relationship between APP‐derived peptides and synapses.

## Author Contributions

I.I.‐G. and A. Lleó contributed to the conception and design of the study; I.I.‐G., J.P., V.M., D.A., E.V., A.B., S.B.‐E, F.S., A.S., M.B.S.‐S., R.R.‐G., R.B., J.C., A.A., A. Lladó, R.‐S.‐V., J.F., and A. Lleó contributed to acquisition of data; I.I.‐G., J.P., V.M., E.V. A.B., F.S., and J.C. contributed to analysis of data and preparing the figures; I.I.‐G., J.P., D.A., J.C., and A. Lleó contributed to drafting the text.

## Conflict of Interests

Nothing to report.

## Supporting information


**Table S1.** Analytical procedures.
**Table S2.** List of genes with the highest confidence score of interaction with APP gene from STRING.
**Table S3.** List of genes with differential expression at cerebral regions where cortical thickness correlated with APP‐derived peptides.
**Table S4.** Gene enrichment analysis
