# Supplementary material for: APP‐derived peptides reflect neurodegeneration in frontotemporal dementia
Source: Ann Clin Transl Neurol. 2019 Dec 2;6(12):2518–30. doi: 10.1002/acn3.50948 (PMC6917306; doi:10.1002/acn3.50948)
Supplement: Supplementary file 1 — Table S1. Analytical procedures. Table S2. List of genes with the highest confidence score of interaction with APP gene from STRING. Table S3. List of genes with differential expression at cerebral regions where cortical thickness correlated with APP‐derived peptides. Table S4. Gene enrichment analysis [file ACN3-6-2518-s001.docx]

**Supplementary Material**

**Sample composition**

The final sample included 214 participants: 93 with FTLD-related syndromes (53 behavioral variant of frontotemporal dementia [bvFTD], 21 non-fluent variant of primary progressive aphasia [nfaPPA], 6 semantic variant of primary progressive aphasia [svPPA] and 13 within progressive supranuclear palsy-corticobasal degeneration spectrum [PSP-CBD]), 57 patients with Alzheimer’s disease (24 at the mild cognitive impairment stage and 33 at the dementia stage), and 64 healthy controls. Participants with FTLD-related syndromes were recruited in two centers as part of the collaborative Catalan Frontotemporal Dementia Initiative (CATFI): the hospital de la Santa Creu i Sant Pau (n=76) and the Hospital Clinic de Barcelona (n=17). Alzheimer’s disease and healthy control participants were recruited from the Sant Pau Initiative on Neurodegeneration cohort and Hospital Clinic de Barcelona (SPIN cohort: <https://santpaumemoryunit.com/our-research/spin-cohort/>). Further details on the clinical protocol of the SPIN cohort can be found elsewhere.^31^ The CATFI is a multicenter study focused on the development of novel biomarkers and therapeutic interventions for patients with frontotemporal dementia. The CATFI study includes patients from three centers (Hospital de Sant Pau [HSP], Hospital Clínic de Barcelona [HCB] and Hospital Arnau de Vilanova).

**Neuropsychological measures**

Cognitive functioning was assessed using a previously described neuropsychological evaluation^1^ close to the MRI acquisition. Four major cognitive domains were covered, similarly to previous studies^2^: memory (the total free recall and the delayed total recall scores of the Free and Cued selective Reminding test, and the delayed recall of the CERAD figures); executive functioning (Digit Span backwards, Trail Making Test part B, and Letter Fluency), language (Category Fluency and Boston Naming Test) and visuo-spatial functioning (Number Location from the Visual Object Space and Perception battery, the Poppelreuter and the copy of CERAD figure). To obtain composite scores for each cognitive domain, we used the means and standard deviations of the healthy control group to convert raw cognitive scores into Z-scores. Subsequently, patients Z-scores were averaged within each cognitive domain. Mini-mental State Examination (MMSE) was available for all clinical groups at both centers and was used as a general measure of cognitive impairment.

**CSF sampling and analyses**

All biomarkers were analyzed at the Sant Pau Memory Unit Laboratory with commercially available ELISA kits of Aβ1-40, Aβ1-38 and sAPPß (EUROIMMUN; EUROIMMUN; IBL, respectively) following previously reported methods and manufacturer’s instructions.^3,4^ For further details on analytical refer to **Supplementary Table 1**. Levels of Aβ1-42 were analyzed with the Lumipulse automated platform (Fujirebio-Europe). To account for inter-center variability in pre-analytical protocols we applied a previously validated harmonization method.^5^ Briefly, the center-driven differences in the distributions of CSF raw measurements were compensated by computing a parametric transformation between the distributions of biomarkers between both sites using a subsampling procedure. Importantly, this previously validated approach preserves relevant biological information such as age, diagnostic group and sex when computing the optimal normalization.

**Supplementary Table 1: Analytical procedures**

|  | Aβ1-42 | Aβ1-40 | Aβ1-38 | sAPPß |
| --- | --- | --- | --- | --- |
| Assay | Lumipulse | EUROIMMUN | EUROIMMUN | IBL |
| Inter-run CV% | 2.8%* | 5.9% | 7.2% | 8.4% |
| Mean intra-assay CV% | Automated platform. Measures in singlets, as recommended by manufacturer | 5.3% | 3.7% | 1.6% |
| Calibrators | 3 levels, as provided by manufacturer | 6 levels, as provided by manufacturer | 6 levels, as provided by manufacturer | 8 levels, as provided by manufacturer |
| Dilution factor | None | 1:21 | None | 1:50 |

**Supplementary Table 1 - Footnotes:** * = performance for AB1-42 in the Lumipulse G600II instrument in our center.^6^

**Cortical gene expression**

Briefly, the probes that do not exceed the background noise in more than 50% of the samples were removed; then, a representative probe for a gene was selected based on the highest intensity value resulting in a total of 15.745 genes sampled.^7^ These preprocessing steps produce a region x gene matrix with the MNI coordinates that can be used for the ROI analysis. Finally, a neuro-related subset was obtained using GO term analysis.^8^ The number of genes in this subset was reduced from 15.745 to 3.605.

**Supplementary Table 2: List of genes with the highest confidence score of interaction with APP gene from STRING.**

| Node1 | Node2 | Coexpression | Experimentally determined interaction | Database annotated | Automated textmining | Combined STRING score |
| --- | --- | --- | --- | --- | --- | --- |
| *APBB1* | *APP* | 0,091 | 0,974 | 0,8 | 0,956 | 0,999 |
| *PSEN1* | *APP* | 0 | 0,461 | 0,9 | 0,974 | 0,998 |
| *PSEN2* | *PSEN1* | 0 | 0,993 | 0,8 | 0,961 | 0,998 |
| *BACE1* | *APP* | 0,089 | 0,406 | 0,9 | 0,958 | 0,997 |
| *APP* | *APOE* | 0,071 | 0,379 | 0,9 | 0,937 | 0,995 |
| *ITM2B* | *APP* | 0,087 | 0,472 | 0,9 | 0,801 | 0,989 |
| *GGA1* | *BACE1* | 0 | 0,379 | 0,9 | 0,778 | 0,985 |
| *SNCA* | *APP* | 0,063 | 0,555 | 0,9 | 0,684 | 0,985 |
| *MAPK8* | *APP* | 0,052 | 0,294 | 0,9 | 0,791 | 0,984 |
| *A2M* | *ALB* | 0,119 | 0 | 0,9 | 0,734 | 0,974 |
| *APLP2* | *ADAM10* | 0,062 | 0,19 | 0,9 | 0,687 | 0,973 |
| *CDH2* | *ADAM10* | 0,077 | 0 | 0,9 | 0,726 | 0,972 |
| *TNFRSF21* | *APP* | 0,088 | 0,723 | 0 | 0,901 | 0,972 |
| *NAE1* | *APP* | 0 | 0,549 | 0,8 | 0,698 | 0,97 |
| *APBA2* | *APP* | 0,175 | 0,864 | 0 | 0,75 | 0,969 |
| *S100B* | *APP* | 0,062 | 0 | 0,9 | 0,686 | 0,968 |
| *INS* | *APP* | 0 | 0 | 0,9 | 0,669 | 0,965 |
| *MAPK10* | *APP* | 0,087 | 0 | 0,9 | 0,637 | 0,964 |
| *TGFB2* | *APP* | 0,116 | 0,524 | 0,9 | 0,233 | 0,963 |
| *GSN* | *APP* | 0,088 | 0,379 | 0,9 | 0,4 | 0,961 |
| *ADAM10* | *APOE* | 0 | 0 | 0,9 | 0,53 | 0,95 |
| *CLU* | *TGFB1* | 0 | 0 | 0,9 | 0,371 | 0,934 |
| *CASP6* | *NGFR* | 0 | 0 | 0,9 | 0,266 | 0,923 |
| *ALB* | *APLP2* | 0,063 | 0 | 0,9 | 0,224 | 0,92 |
| *SORL1* | *APOE* | 0 | 0,305 | 0,54 | 0,733 | 0,907 |

**Supplementary Table 2 - Footnotes:** List of genes with the highest confidence score of interaction from STRING.

**Supplementary Table 3: List of genes with differential expression at cerebral regions where cortical thickness correlated with APP-derived peptides**

| Gene | z-score | t-value | p-value | Effect size |
| --- | --- | --- | --- | --- |
| *PRRX1* | -4,9906294 | -8,6358083 | 2,97E-17 | -0,6535382 |
| *ZIC1* | -4,1471097 | -7,3244634 | 5,72E-13 | -0,5548797 |
| *FZD10* | -3,5216638 | -6,3671664 | 3,19E-10 | -0,4817273 |
| *ANXA1* | -3,4600917 | -6,2664341 | 5,93E-10 | -0,4745258 |
| *BRINP3* | -3,4155181 | -6,1769172 | 1E-09 | -0,4693124 |
| *MCTP2* | -3,2689194 | -5,9733345 | 3,45E-09 | -0,4521662 |
| *CDH13* | -3,1307374 | -5,7537788 | 1,23E-08 | -0,4360043 |
| *NGB* | -3,0884647 | -5,6865476 | 1,8E-08 | -0,4310601 |
| *CTSH* | -3,0581203 | -5,6332585 | 2,43E-08 | -0,427511 |
| *ITGB4* | -3,0416717 | -5,6266007 | 2,51E-08 | -0,4255872 |
| *OSTN* | -3,0367057 | -5,5937769 | 3,02E-08 | -0,4250064 |
| *EPHB2* | -2,9921478 | -5,543675 | 3,98E-08 | -0,4197948 |
| *SPTBN5* | -2,9762224 | -5,5141229 | 4,68E-08 | -0,4179322 |
| *SYT6* | -2,9489016 | -5,4887976 | 5,37E-08 | -0,4147367 |
| *ADCYAP1* | -2,9372431 | -5,4455535 | 6,82E-08 | -0,4133732 |
| *CNTNAP2* | -2,9342852 | -5,4714674 | 5,92E-08 | -0,4130272 |
| *P2RX6* | -2,8608631 | -5,3572501 | 1,09E-07 | -0,4044397 |
| *NXPH3* | -2,812146 | -5,2568835 | 1,87E-07 | -0,3987418 |
| *SYT10* | -2,7680675 | -5,1875782 | 2,68E-07 | -0,3935863 |
| *HES1* | -2,7598205 | -5,1834993 | 2,74E-07 | -0,3926217 |
| *NREP* | -2,7350714 | -5,1439528 | 3,36E-07 | -0,3897271 |
| *GDPD5* | -2,7331563 | -5,1553867 | 3,17E-07 | -0,3895031 |
| *ANGPT1* | -2,6913508 | -5,0680032 | 4,96E-07 | -0,3846135 |
| *ONECUT2* | -2,6511013 | -5,0169142 | 6,43E-07 | -0,3799059 |
| *KCNA4* | 2,63329012 | 3,13900239 | 0,00175549 | 0,23815899 |
| *MYO7A* | 2,63895006 | 3,13718651 | 0,00176656 | 0,23882098 |
| *ERC2* | 2,64789981 | 3,16679934 | 0,00159773 | 0,23986774 |
| *WNT7B* | 2,7154304 | 3,26510898 | 0,00113955 | 0,24776615 |
| *PDPK1* | 2,73179558 | 3,28915165 | 0,00104754 | 0,24968023 |
| *EPHA6* | 2,75496455 | 3,32100882 | 0,00093639 | 0,25239009 |
| *KIRREL3* | 2,76747956 | 3,34672782 | 0,00085462 | 0,25385385 |
| *ENC1* | 2,7700546 | 3,34983465 | 0,00084526 | 0,25415503 |
| *SYN3* | 2,79035935 | 3,38888407 | 0,00073502 | 0,25652988 |
| *RAB3D* | 2,79040658 | 3,37468103 | 0,0007736 | 0,2565354 |
| *FBXO2* | 2,80059932 | 3,39817192 | 0,00071061 | 0,25772755 |
| *MOXD1* | 2,80095639 | 3,41443876 | 0,00067006 | 0,25776931 |
| *GRM8* | 2,85997864 | 3,50278642 | 0,00048493 | 0,26467258 |
| *NPTXR* | 2,87992922 | 3,52142626 | 0,00045281 | 0,26700601 |
| *LINGO3* | 2,88205604 | 3,53018729 | 0,00043813 | 0,26725477 |
| *NPTX1* | 2,96952971 | 3,65122323 | 0,00027746 | 0,27748573 |
| *USP46* | 2,99671151 | 3,70965135 | 0,0002214 | 0,28066492 |
| *DRD2* | 3,01202601 | 3,74047566 | 0,00019628 | 0,28245611 |
| *CPLX2* | 3,04579201 | 3,78650699 | 0,0001638 | 0,2864054 |
| *EGR3* | 3,04588659 | 3,7716439 | 0,00017373 | 0,28641646 |
| *GRK5* | 3,05984923 | 3,79657388 | 0,00015744 | 0,28804954 |
| *PLPPR5* | 3,07479163 | 3,81420403 | 0,00014681 | 0,28979721 |
| *BDKRB2* | 3,16674692 | 3,96136435 | 0,000081 | 0,30055234 |
| *HEY1* | 3,18406938 | 4,00032671 | 0,0000689 | 0,30257839 |
| *PDYN* | 3,24594473 | 4,07187529 | 0,0000511 | 0,30981536 |
| *HTR4* | 3,62835853 | 4,66875618 | 0,00000353 | 0,35454265 |
| *SORCS1* | 3,83482784 | 4,98033114 | 7,74E-07 | 0,3786914 |
| *EPCAM* | 3,84907681 | 5,01837507 | 6,38E-07 | 0,38035797 |
| *NR2F2* | 3,95309063 | 5,16757757 | 2,98E-07 | 0,39252347 |
| *LAMP5* | 4,09351806 | 5,38518609 | 9,44E-08 | 0,40894793 |
| *MET* | 4,12431664 | 5,44893593 | 6,7E-08 | 0,41255014 |
| *NPTX2* | 4,2272108 | 5,59324791 | 3,03E-08 | 0,42458469 |
| *SLC17A6* | 4,28838026 | 5,69846373 | 1,68E-08 | 0,4317391 |

**Supplementary Table 3 - Footnotes:** List of genes with differential expression at cerebral regions where cortical thickness correlated with APP-derived peptides

**Supplementary Table 4:**

| GO biological process | Rank in gene list | Fold Enrichment | Raw *p* value | FDR |
| --- | --- | --- | --- | --- |
| Synaptic signaling (GO:0099536) | 439 | 11,75 | 1,24E-11 | 2,17E-08 |
| Trans-synaptic signaling (GO:0099537) | 429 | 11,16 | 1,37E-10 | 0,00000018 |
| Anterograde trans-synaptic signaling (GO:0098916) | 411 | 10,75 | 1,16E-09 | 0,00000102 |
| Chemical synaptic transmission (GO:0007268) | 411 | 10,75 | 1,16E-09 | 0,00000108 |
| Modulation of chemical synaptic transmission (GO:0050804) | 438 | 9,25 | 2,8E-08 | 0,0000177 |
| Regulation of trans-synaptic signaling (GO:0099177) | 439 | 9,23 | 2,87E-08 | 0,0000174 |
| Neuron projection development (GO:0031175) | 659 | 8,38 | 2,07E-10 | 2,51E-07 |
| Neuron development (GO:0048666) | 804 | 7,33 | 3,15E-10 | 3,32E-07 |
| Cell-cell signaling (GO:0007267) | 1093 | 7,08 | 4,48E-13 | 1,77E-09 |
| Neuron differentiation (GO:0030182) | 992 | 7,06 | 8,43E-12 | 1,9E-08 |
| Generation of neurons (GO:0048699) | 1513 | 6,09 | 3,26E-14 | 2,58E-10 |
| Neurogenesis (GO:0022008) | 1612 | 5,94 | 1,39E-14 | 2,19E-10 |
| Plasma membrane bounded cell projection organization (GO:0120036) | 1082 | 5,79 | 2,7E-09 | 0,00000194 |
| Cell projection organization (GO:0030030) | 1122 | 5,58 | 4,62E-09 | 0,00000305 |
| Nervous system development (GO:0007399) | 2324 | 4,6 | 1,38E-13 | 7,26E-10 |
| Cell development (GO:0048468) | 1603 | 4,14 | 0,00000014 | 0,000079 |
| Anatomical structure morphogenesis (GO:0009653) | 2102 | 3,68 | 6,38E-08 | 0,0000373 |
| Cellular developmental process (GO:0048869) | 3734 | 3,35 | 2,39E-12 | 7,55E-09 |
| Cell differentiation (GO:0030154) | 3642 | 3,34 | 7,82E-12 | 2,06E-08 |
| Regulation of cell communication (GO:0010646) | 3634 | 3,04 | 1,52E-09 | 0,00000127 |
| Regulation of signaling (GO:0023051) | 3674 | 3,01 | 1,99E-09 | 0,00000157 |
| System development (GO:0048731) | 4367 | 2,95 | 3,6E-11 | 5,17E-08 |
| Signaling (GO:0023052) | 5293 | 2,71 | 9,7E-12 | 1,92E-08 |
| Multicellular organism development (GO:0007275) | 4963 | 2,67 | 2,7E-10 | 3,05E-07 |
| Cell communication (GO:0007154) | 5401 | 2,66 | 1,89E-11 | 2,99E-08 |
| Anatomical structure development (GO:0048856) | 5342 | 2,48 | 2,34E-09 | 0,00000176 |
| Developmental process (GO:0032502) | 5686 | 2,4 | 2,93E-09 | 0,00000201 |
| Multicellular organismal process (GO:0032501) | 6886 | 2,25 | 4,62E-10 | 4,57E-07 |

**Supplementary Table 4 - Footnotes:** Gene enrichment analysis (FDR < 0.0001). GO processes with FDR < 0.0001 are shown.

**Abbreviations:** GO = gene ontology; FDR = false discovery rate.

**Supplementary references**

1. Sala I, Illán-Gala I, Alcolea D, et al. Diagnostic and Prognostic Value of the Combination of Two Measures of Verbal Memory in Mild Cognitive Impairment due to Alzheimer’s Disease. J Alzheimers Dis 2017;58(3):909–918.

2. Ossenkoppele R, Pijnenburg YAL, Perry DC, et al. The behavioural/dysexecutive variant of Alzheimer’s disease: clinical, neuroimaging and pathological features. Brain 2015;138(Pt 9):2732–2749.

3. Alcolea D, Martínez-Lage P, Sánchez-Juan P, et al. Amyloid precursor protein metabolism and inflammation markers in preclinical Alzheimer disease. Neurology 2015;85(7):626–633.

4. Vanderstichele HMJ, Janelidze S, Demeyer L, et al. Optimized Standard Operating Procedures for the Analysis of Cerebrospinal Fluid Aβ42 and the Ratios of Aβ Isoforms Using Low Protein Binding Tubes. J Alzheimers Dis 2016;53(3):1121–1132.

5. Zhou HH, Singh V, Johnson SC, et al. Statistical tests and identifiability conditions for pooling and analyzing multisite datasets. Proc. Natl. Acad. Sci. U.S.A. 2018;115(7):1481–1486.

6. Alcolea D, Pegueroles J, Muñoz L, et al. Agreement of amyloid PET and CSF biomarkers for Alzheimer’s disease on Lumipulse. Ann Clin Transl Neurol 2019;6(9):1815–1824.

7. Arnatkevic Iūtė A, Fulcher BD, Fornito A. A practical guide to linking brain-wide gene expression and neuroimaging data. Neuroimage 2019;189:353–367.

8. Diez I, Sepulcre J. Neurogenetic profiles delineate large-scale connectivity dynamics of the human brain. Nat Commun 2018;9(1):3876.
